# Jarring

**Published:** 1890-07

**Authors:** 


Jarring or rattling of wagons head sensitive to, also to stepping hard, Nit-ac. Abdomen sensitive to jarring, Lil-tig. Jarring when sitting in a chair or lying in bed, Aloes. Sensitiveness to, and aggravation from jarring the bed is the great key-note of Belladonna.

